# Obesity and the obesity paradox in abdominal aortic aneurysm

**DOI:** 10.3389/fendo.2024.1410369

**Published:** 2024-07-11

**Authors:** Feng Lu, Yong Lin, Jianshun Zhou, Zhen Chen, Yingying Liu, Maolin Zhong, Lifeng Wang

**Affiliations:** ^1^ Department of Anesthesiology, The First Affiliated Hospital of Gannan Medical University, Ganzhou, Jiangxi, China; ^2^ Ganzhou Key Laboratory of Anesthesiology, The First Affiliated Hospital of Gannan Medical University, Ganzhou, Jiangxi, China; ^3^ Key Laboratory of Prevention and Treatment of Cardiovascular and Cerebrovascular Diseases, Ministry of Education, Gannan Medical University, Ganzhou, Jiangxi, China

**Keywords:** abdominal aortic aneurysm, obesity, obesity paradox, prehabilitation, aging

## Abstract

Obesity, characterized by its complexity and heterogeneity, has emerged as a significant public health concern. Its association with increased incidence and mortality of cardiovascular diseases stems not only from its complications and comorbidities but also from the endocrine effects of adipose tissue. Abdominal aortic aneurysm (AAA), a chronic inflammatory condition, has been closely linked to obesity. Intriguingly, mild obesity appears to confer a protective effect against AAA mortality, whereas severe obesity and being underweight do not, giving rise to the concept of the “obesity paradox”. This review aims to provide an overview of obesity and its paradoxical relationship with AAA, elucidate its underlying mechanisms, and discuss the importance of preoperative weight loss in severely obese patients with AAA.

## 1 Introduction

Obesity, characterized by abnormal or excessive fat accumulation, constitutes not only a metabolic disorder but also a chronic inflammatory, degenerative, and psychosocial ailment that poses a significant threat to public health (1–4). Particularly, central obesity has been strongly associated with the development of cardiovascular diseases and increased mortality (5–7).

Abdominal aortic aneurysm (AAA), likened to an untimed bomb within the body, is defined as a 50% dilation of the abdominal aorta, typically exceeding 3 cm. It is recognized as a chronic inflammatory degenerative disorder of the aorta (8). Risk factors for AAA encompass aging, hypertension, hyperlipidemia, smoking, and a family history of AAA (9). While obesity traditionally isn’t regarded as a risk factor for AAA, there’s a growing body of literature suggesting a correlation between weight gain and a heightened incidence of AAA and associated mortality. However, studies regarding the impact of obesity on AAA present conflicting findings (10).

Obesity encompasses not only the accumulation of fat cells in the body but, more significantly, is often accompanied by adverse lifestyle choices and various comorbidities. These factors collectively influence the initiation, progression, and prognosis of AAA. Consequently, analyzing obesity as a singular variable leads to complexity and inaccuracies in clinical observations and outcomes.

In this review, our aim is to explore the intricate relationship between obesity and AAA, elucidating the underlying mechanisms. Additionally, given the elevated risk of adverse events during the perioperative period, perioperative weight loss in obese patients has garnered considerable attention. Therefore, we intend to summarize the current published research on the timing and methods of weight loss, aiming to delineate the potential role of intentional weight loss in the prevention and management of AAA, and to inspire future research (**Table 1**).

**Table 1:** The relationship between obesity and abdominal aortic aneurysm.

The relationship between obesity and abdominal aortic aneurysm morbidity							
Obesity Index	Definition	Obesity Measure	Subjects	Abdominal Aortic Segments	Aortic Diameter Measurement Method	Relationship with AAA	Ref
Body Mass Index (BMI)	Weight (kg)/Height^2^ (m^2^)	Overall level of obesity	Human (504 participants)	Superior mesenteric artery (SMA) segment; Aortic midpoint segment; Aortic bifurcation segment	Computerized Tomography (CT)	BMI is positively associated with increasing aortic diameter at the SMA segment (standardized β=0.68 p<0.01)	(10)
			Human (3,056,455 participants)	The greater of orthogonal and transverse ultrasound measurements of the infrarenal abdominal aorta	Ultrasound	BMI>25 is significantly associated with AAA prevalence (OR=1.20[95% CI, 1.17-1.22], P<0.001)	(11)
Fat-free Mass Index	Assessed using bioelectrical impedance Technique, fat-free mass (kg)/Height^2^ (m^2^)	The percentage of the body mass corresponding to fat-free mass	Human (367703 participants)	Not available	Not available	There was significant evidence that fat-free mass index was inversely associated with abdominal aortic aneurysm [OR=0.64 (95% CI, 0.42-0.95), P=0.03].	(12)
Waist circumference (WC); Waist-to-hip ratio (WHR)	WC is measured at the level between the lowest rib and iliac bone; WHR is the WC divided by the hip circumference	Central (abdominal) obesity	Human (12203 participants)	The maximum diameter of the infrarenal aorta	Ultrasound	Waist circumference [OR=1.14 (95% CI, 1.06-1.22), P<0.001] and waist-to-hip ratio [OR=1.22 (95% CI, 1.09-1.37), P<0.001] were independently associated with AAA and aortic diameter, especially for AAA ≥40 mm [OR=1.53 for waist-to-hip ratio (95% CI, 1.26-1.85), P<0.001].	(13)
Fat Percentage (BF%)	Measured by hydrostatic weighing, predicted by body skinfolds measurements or by DXA whole body scans	The percentage of the body mass corresponding to fat mass	Human (504 participants)	Superior mesenteric artery segment; Aortic midpoint segment; Aortic bifurcation segment	Computerized Tomography (CT)	Positively associated with increasing aortic diameter at the SMA segment (standardized β=0.12 p<0.01)	(10)
Periaortic Adipose Tissue Volume	The volume of adipose tissue at mid–abdominal aorta	ectopic fat depots and central obesity	Human (3000 participants)	A level 5 cm above the aortoiliac bifurcation	CT	Periaortic fat volume was associated with aortic dimensions in abdomen (P<0.001)	(14)
PVAT Density	The ratio between the number of fat pixels inside ROI and the area of the ring	Ectopic fat depots	Human (341 participants)	The maximum length between opposite walls on AAA main axis	CT	Individual PVAT differences were positively correlated with aortic volume. (P=0.006) The presence of aneurysms was an independent predictor of increased differences in PVAT *in vivo*	(15)
Epicardial Adipose Tissue volume (EATV)	The volume of visceral adipose tissue that is located between the pericardium and myocardium	Ectopic fat depots	Human (237patients with infrarenal and fusiform AAA)	The difference between the initial and last maximal short- axis diameter of AAA	CT	AAA expansion rate is positively correlated with EATV index (R =.237, P <.001). Initial aneurysm diameter and EATV index > 60.3 cm^3^/m^2^ were significantly correlated with AAA dilation rate (P<0.001)	(16)
Intramuscular Fat	Using threshold method with semiautomatic segmentation procedure	Ectopic fat depots; Associated with postoperative recovery	Human (94 AAA patients undergone EVAR)	Difference in aneurysm volume before and after EVAR; Maximum aneurysm diameter	CT	Patients with more intramuscular fat had a high risk of aneurysm dilation.	(17)
The relationship between obesity and abdominal aortic aneurysm mortality							
Body Mass Index (BMI)	Weight (kg)/Height^2^ (m^2^)	Overall level of obesity	Human (33082 AAA patients)	Not available	Not available	There is no significant difference in mortality between the obesity and non-obesity groups undergoing OAR and EVAR [OAR, 2.9% vs 3.2% (P = .50); EVAR, 0.5% vs 0.6% (P = .76)]. However, risk of renal failure and wound infections is higher in obese group.	(18)
			Human (33082 patients with pararenal and thoracoabdominal aortic aneurysms)	Not available	Not available	There exist no significant differences between the obese and nonobese patients undergoing FB-EVAR in 3-years survival (83% ± 5% vs 75% ± 4%; P = 0.16), although 2 and 12 months QOL scores showed higher in the obese patients.	(19)
			Human (80 AAA patients undergoing elective EVAR)	Not available	Not available	Obesity(BMI≥30) have little influence on outcomes after EVAR during 2 years of follow-up (p = 0.688).	(20)
			Human (2201 AAA patients)	Not available	Not available	Obesity(BMI≥30) independently predicts increased cardiac (OR=4.5 for obesity III, P=0.045) and renal complications (OR=6.3 for obesity III, P<0.001) after open AAA repair and predicts increased wound complications after both open repair(OR=2.4 for obesity I, P=0.002) and EVAR(OR=3.1 for obesity I, P=0.026), despite no statistically difference in 30 day mortality.	(21)
			Human (103,972 AAA patients)	Not available	Not available	BMI is positively associated with AAA mortality among men patients [HR=3.81(95% CI, 1.39–10.49), P=0.01]	(22)
			Human (202 patients with ruptured AAA)	Not available	Not available	For every 1 kg/m2 increase in BMI, the risk of 30-day death in hospitalized patients with rAAA repair was significantly increased by 1.08 (95% CI, 1.01-1.17; P =0.04).	(23)
			Human (5455 AAA patients)	Not available	Not available	The 30-day mortality rates of OAR (7.3%) and EVAR (2.4%) were higher in morbidly obese patients than in obese patients (3.9%; 1.5%) or non-obese (3.7%; 1.1%).	(24)
			Human	Not available	Not available	Trends in BMI were negatively and significantly associated with AAA mortality in both male and female (P=0.0039).	(25)
			Human (7543 AAA patients)	Not available	Not available	Class I obese (BMI 25.1-30) patients have a significantly lower 30-day risk of death compared to normal weight patients (P<0.05),	(26)

## 2 Obesity to AAA: foe or friend?

Although obesity is not explicitly designated as a risk factor for AAA in current guidelines, several clinical studies have suggested that excessive fat accumulation does indeed heighten the risk of developing AAA. For instance, a case-control study involving 504 participants revealed a positive correlation between BMI and increasing aortic diameter at the superior mesenteric artery (SMA) segment (10) (**Table 1**). Additionally, various methods of evaluating obesity have shown a corresponding increase in AAA incidence with the severity of obesity.

When stratifying obese patients based on the degree of obesity, an intriguing trend emerges concerning the perioperative mortality of AAA. A retrospective study involving 5,455 AAA patients revealed that morbidly obese individuals (BMI>34 kg/m^2^) exhibited higher 30-day mortality rates in both open aneurysm repair (OAR) and endovascular aneurysm repair (EVAR) procedures compared to non-obese patients, including those with milder obesity (24). Additionally, for every 1 kg/m2 increase in BMI, the risk of 30-day death in hospitalized patients undergoing repair for ruptured abdominal aortic aneurysm was significantly elevated by 1.08 (95% CI,1.01-1.17; P=0.04) (23).

However, patients classified as mildly obese displayed the lowest mortality rates post-AAA surgery, even lower than those with normal weight (25, 26). Further investigation through a retrospective analysis of 9,479 patients undergoing OAR revealed that individuals categorized as overweight (BMI: 25-30 kg/m^2^) or mildly obese (BMI>30 kg/m^2^) did not exhibit additional surgical mortality associated with BMI until BMI surpassed 34 kg/m^2^ (27). Interestingly, mortality in AAA patients does not correspondingly increase with the severity of obesity; it is lowest in those with mild obesity but elevated in patients with low weight or excessive obesity. Although certain studies have concluded that obesity does not exert a statistically significant effect on the perioperative mortality of AAA, this may stem from a lack of BMI stratification among patients. Nevertheless, these studies have consistently indicated that both low body weight and severe obesity predispose individuals to increased perioperative complications such as renal insufficiency and wound infections, thereby contributing to poorer prognoses in AAA patients (18–21). This phenomenon, akin to the “obesity paradox” observed in other cardiovascular diseases, underscores the complex interplay between obesity and AAA outcomes (28–30).

Given the inconsistent role of obesity in the epidemiology and prognosis of AAA, we will address on the following aspects

## 3 Potential mechanism of obesity aggravating AAA

As outlined in Section 2 and corroborated by **Table 1**, it is evident that obesity augments the incidence of AAA. Numerous clinical studies have substantiated a positive correlation between the degree of obesity and the diameter of the abdominal aorta in AAA patients, irrespective of the method used to evaluate obesity (10, 13, 16, 17).

Obesity, recognized as a chronic degenerative disease, manifests cardiovascular implications at an earlier age compared to individuals with normal weight, essentially reflecting accelerated systemic aging (31). Senescent cells accumulate with aging, obesity, and diabetes, particularly within adipose tissue depots, encompassing subcutaneous, visceral, and intramuscular spaces (8, 32). Excessive energy intake disrupts the delicate energy balance, leading to hypertrophy and hyperplasia of adipose tissue around various organs, including the liver, heart, muscle, kidney, pancreas, and inducing insulin resistance (33). Moreover, obesity, hypercholesterolemia, hypertension, and high-fat diets contribute to an inflammatory milieu, oxidative/nitrification stress, mitochondrial dysfunction, endothelial apoptosis, macromolecular damage, and vascular wall senescence, consequently heightening cardiovascular risk and disease, including AAA (34).

Similarly, AAA, characterized as a chronic inflammatory degenerative ailment, essentially represents vascular senescence, with its prevalence escalating with age (35, 36). Experimental studies have delineated transcriptional alterations in abdominal aortic tissue during aging-induced AAA, characterized by smooth muscle cell loss, leukocyte adhesion, inflammation, and accumulation of senescent cells in the vascular wall and perivascular adipose tissue (PVAT) (37). Pathological changes observed in human AAA closely resemble those occurring in the abdominal aorta due to obesity (38). Furthermore, experiments have underscored the impact of preaging adipocyte secretions on vascular wall cell phenotype and function, mirroring observations in aneurysmal vessel walls (39).

### 3.1 Adipose tissue in the pathological state results in high AAA morbidity and mortality

As obesity progresses, human adipocytes not only undergo hypertrophy and hyperplasia but also transition from a physiological to a pathological state, thereby contributing to endocrine disorders associated with severe obesity (40). The adipose tissue in the human body comprises two main categories, primarily subcutaneous adipose tissue (SAT) and visceral adipose tissue (VAT). Predominantly composed of white adipocytes and often indicated by waist circumference, VAT is strongly linked to dyslipidemia and hypertension. Clinical studies have consistently demonstrated a significant association between increased VAT and a heightened risk of incident AAA (40–42).

Additionally, there exists a correlation between visceral abdominal adiposity and the volume and density of abdominal periaortic adipose tissue, which is directly related to the size of the aorta (14, 15, 43). This relationship may stem from the direct action of adipose tissue in its pathological state on the abdominal aorta, which can be concluded as an aging process. Key mechanisms include:

Firstly, obesity enhances the production of reactive oxygen species (ROS), resulting in oxidative stress. This oxidative damage accelerates endothelial dysfunction and promotes vascular stiffness, both of which are hallmarks of vascular aging and can eventually lead to AAA (44).

Moreover, adipose tissue in obese individuals secretes pro-inflammatory cytokines such as TNF-α, IL-6, and MCP-1. These cytokines induce a chronic inflammatory state that contributes to vascular aging by promoting endothelial cell apoptosis and reducing the regenerative capacity of vascular cells, processes that have been shown to cause AAA (45, 46).

**Figure 1:**
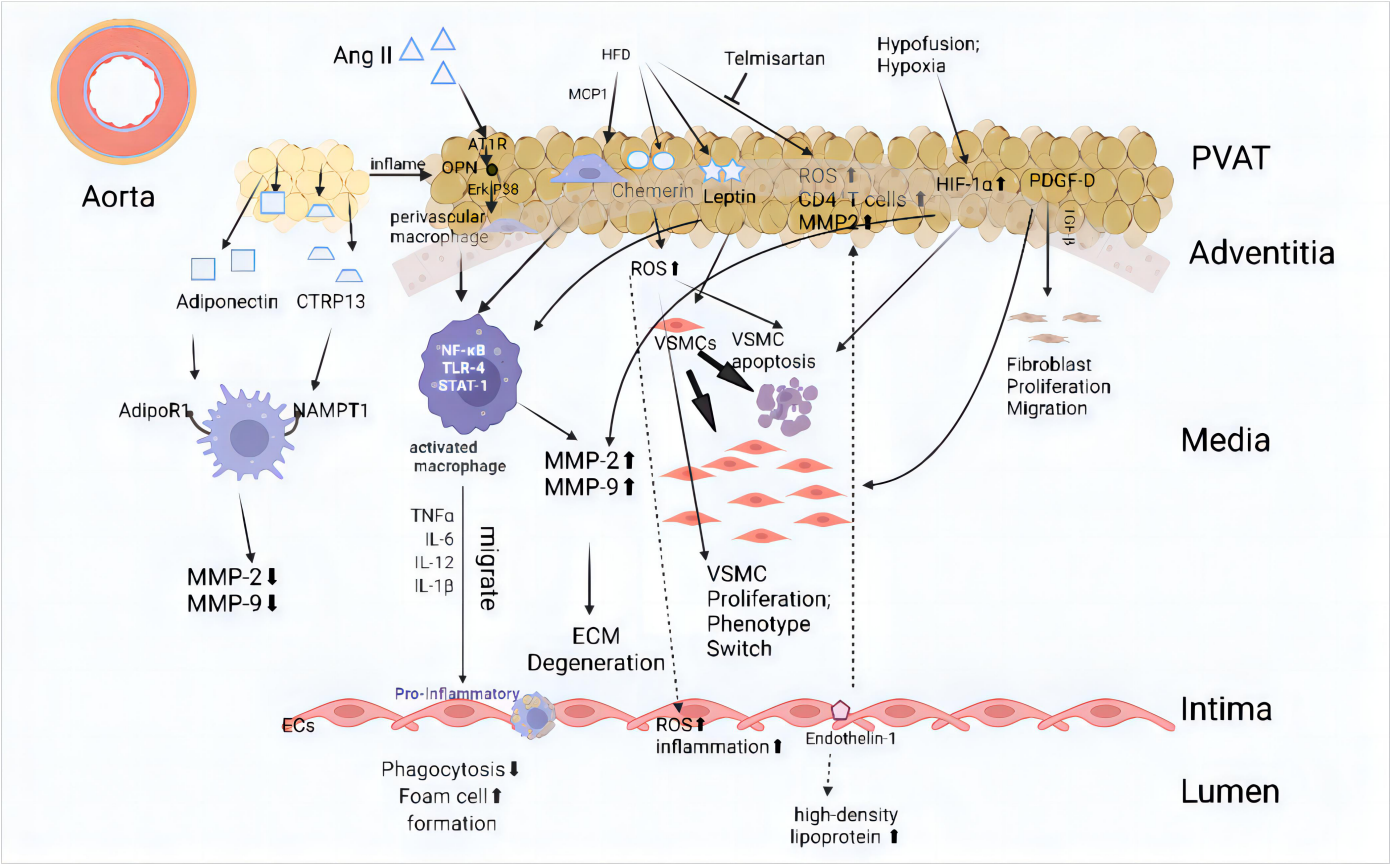
Mechanism of adipose tissue action on all layers of the abdominal aorta Internal or external environmental changes such as hypoxia and a high-fat diet can impact adipose tissue, leading to pathological changes and the release of adipokines. In a physiological state, perivascular adipose tissue (PVAT) can cause a full-thickness lesion of the arterial wall from the outside to the inside. The main manifestations include the aggregation of inflammatory cells, increased reactive oxygen species (ROS), changes in vascular smooth muscle cell (VSMC) phenotype, endothelial cell dysfunction, and extracellular matrix (ECM) degradation. These changes ultimately exacerbate abdominal aortic aneurysm (AAA). The main pathways include (1): Angiotensin II (Ang II): Ang II interacts with AT1R, triggering the OPN and Erk1/2β pathways, which leads to macrophage activation. This activation promotes the release of pro-inflammatory cytokines such as TNFα, TLR-4, and STAT-1, eventually resulting in ECM degeneration and increased endothelial cell inflammation (58–60) (2). High-Fat Diet (HFD): HFD induces the secretion of chemerin, leptin, and MCP1, which promote VSMC proliferation, apoptosis, and phenotype switching (57, 59, 61). Additionally, this process increases ROS and inflammatory responses within the vascular wall (3). Hypoxia and Hypofusion: Conditions like hypoxia induce HIF-1α expression, promoting VSMC apoptosis and the release of MMP-2 and MMP-9. These changes further contribute to ECM degeneration and fibroblast proliferation and migration (62, 63). AT1R, Angiotensin II type 1a receptor; CTRP13, C1q/tumor necrosis factor (TNF)-related protein-13; ECM, Extracellular Matrix; HFD, High-fat diet; MCP-1, Monocyte chemoattractant protein-1; MMP, Matrix metalloproteinase; NAMPT1, Nicotinamide Phosphoribosyl-Transferase 1; OPN, Osteopontin; PDGF-D, Platelet-derived Growth Factor D; TGF-β, Transforming Growth Factor-β; VSMC, vascular smooth muscle cell. (This figure is supported by Biorender).

Furthermore, alterations in adipokines derived from adipose tissue that contribute to aortic remodeling in both AAA patients and experimental models of AAAs can be considered manifestations of vascular aging (37). Some adipokines, such as leptin, and adiponectin, have been strongly associated with AAA diameter (13, 47). Leptin, a highly secreted adipocytokine by PVAT, is considered a proinflammatory factor that elevates levels of cytokines such as TNF-α, IL-6, and IL-12 (48–50). Research suggests that leptin from PVAT promotes AAA formation through IL-18-mediated smooth muscle cell loss, apoptosis, and induced vascular remodeling (51, 52). Pathological PVAT may also exacerbate endothelial dysfunction in diet-induced obese mice due to increased NADPH oxidase-derived oxidative stress and the production of proinflammatory cytokines (53). Additionally, resistin, omentin and vaspin have been shown to be deposited and induce endothelial cell dysfunction through paracrine signaling and other mechanisms (54). Another adipokine derived from PVAT, chemerin, has been shown to exacerbate experimental AAA by inducing endothelial dysfunction via targeting NAD(P)H oxidase in high-fat diet mice (55–57) (**Figure 1**).

### 3.2 Comorbidities and complications along with severe obesity results in high AAA morbidity and mortality

A previous study indicated that the risk of cardiovascular disease associated with a high BMI or increased waist circumference is primarily mediated by altered intermediate risk factors, such as atherosclerotic dyslipidemia and hypertension (12, 64). These factors may collectively contribute to the elevated morbidity of AAA. Lipid accumulation products have emerged as a stronger prognostic marker for all-cause cardiovascular morbidity and mortality compared to BMI, and have been associated with atherosclerosis, as evidenced in a retrospective study involving 9,180 participants (65). Additionally, both a prospective study and a multicenter retrospective analysis have independently confirmed a significant association between increased levels of lipoprotein(a) and the occurrence of AAA (66, 67). Furthermore, most AAAs are considered to represent end stages of atherosclerosis which is positively associated with lipoprotein level (68). Compensatory dilated remodeling, an essential component of atherosclerosis, is intended to delay the development of overt luminal compromise (69).

Additionally, findings from the Framingham Heart Study indicate that 60 to 70% of cases of essential hypertension can be attributed to obesity (70). Moreover, researchers suggest that obesity, characterized by elevated levels of leptin and decreased levels of adiponectin, may disrupt blood pressure regulation (71, 72).This imbalance in blood pressure regulation, in turn, weakens the arterial wall and increases arterial pressure, ultimately leading to arterial deformation and changes in hemodynamics, which can contribute to the progression of AAA (73–75).

Furthermore, individuals with abdominal obesity often lead unhealthy lifestyles, characterized by higher rates of smoking and lower levels of physical activity. These lifestyle factors also play a significant role in the onset and progression of AAA (76, 77).

## 4 The obesity paradox in AAA — is obesity a true friend?

When regression analyses were conducted to identify risk factors related to global AAA mortality, BMI exhibited a negative linear association with AAA mortality (P ≤ 0.007), despite some studies presenting differing conclusions (18, 20, 25). However, upon stratification based on the degree of obesity, the protective effect of overweight or mild obesity on the prognosis of AAA was revealed, contrary to its epidemiological effect on AAA incidence. For instance, a case-control study involving 7,543 patients with AAA found that class I obese individuals (BMI 25.1-30 kg/m^2^) had a significantly lower 30-day risk of death compared to normal weight patients (P<0.05) (26).

Furthermore, a meta-analysis of 92,525 patients undergoing vascular surgery corroborated these findings, showing that patients with a BMI of 25-29.9 kg/m2 exhibited the lowest overall mortality and had fewer cardiac and respiratory complications 30 days after surgery (30). Importantly, this phenomenon extends beyond OAR to EVAR. A meta-study of 14,971 patients undergoing AAA surgery (including 11,743 EVAR cases) confirmed that obese patients had lower 30-day mortality (1.5%) compared to nonobese patients (2.2%) undergoing EVAR (78).This phenomenon is referred to as the obesity paradox, and possible mechanisms include:

As a chronic inflammatory disease, AAA exhibits high metabolic dynamics (79). Weight gain and overall obesity are often indicative of a positive long-term energy balance, where energy intake surpasses energy expenditure, thereby counteracting the energy expenditure associated with chronic inflammation and delayed vascular aging (80). Research from heart failure has shed light on the obesity paradox, which can be attributed to impaired fatty acid oxidation, characterized by mitochondrial dysfunction, reduced availability of essential cofactors such as coenzyme A or carnitine, downregulation of β-oxidase, and increased dependence on alternative substrates such as glucose and ketone bodies (81).The concept of supporting myocardial energetics by increasing fat intake has emerged as an important adjunctive treatment for heart failure.

Galectin 1, a member of the lectin family highly expressed in adipose tissue, has been found to exacerbate obesity in high-fat diet mice by increasing PPARγ expression and activation (82).However, it has also been found to be protective against AAA and atherosclerosis (83).This protective effect is attributed to the attenuation of foam cell formation and mitochondrial dysfunction in vascular smooth muscle cells (VSMCs), as well as the increase in systolic VSMCs in aortic tissue. The secretory function of adipose tissue can further reduce extracellular matrix fibrosis and macrophage infiltration/activation, while enhancing angiogenic potential, thus potentially playing a protective role in the cardiovascular system. However, the specific mechanisms underlying these effects require further exploration (84).

However, obesity, being a multifaceted disease, not only presents numerous comorbidities but also exerts a multifaceted impact on AAA mortality. The current study primarily utilized BMI as an indicator of obesity evaluation to explore its relationship with AAA, without considering the impact of other medical and medication histories. Therefore, whether obesity can be considered a true friend remains a complex and nuanced question that warrants further investigation.

### 4.1 Obesity paradox caused by previous history – medical and medication history

The potential mechanism underlying the obesity paradox may indeed be rooted in timely lifestyle management and medication for obesity-related complications. Often regarded as a silent killer, obese patients with AAA are initially diagnosed and treated for obesity-related comorbidities and complications.

Prospective trials have demonstrated a positive correlation between the incidence of AAAs and BMI as well as waist circumference, similarly observed with diabetes (85, 86). However, a threshold for AAA incidence is noted as waist circumference increases, attributed to the protective effect of diabetes on AAA (42, 87, 88). Despite diabetes being a recognized risk factor for cardiovascular disease, it exhibits an inverse association with the onset, development, and mortality of AAA (87–90). Notably, the protective effect of diabetes is heightened when obesity is controlled (87). One explanation for this phenomenon lies in the usage of hypoglycemic agents, such as metformin and Dapagliflozin (an SGLT-2 inhibitor), which exert anti-inflammatory and anti-aging effects, influencing mechanisms involved in the formation of both experimental and clinical AAA (91–93).

Furthermore, while dyslipidemia and atherosclerosis promote the occurrence and incidence of AAA, vascular calcification has been shown to stabilize the aortic aneurysm wall, potentially preventing AAA dilatation and reducing the risk of rupture and mortality (94, 95). It is demonstrated that established atherosclerosis accelerates AAA growth is limited (96). This discrepancy may stem from different phenotypes between substantial atherosclerosis and clinically insignificant atherosclerosis in AAA patients (8). Moreover, the use of lipid-lowering drugs, such as statins, may contribute to this phenomenon. Nevertheless, calcification has been associated with medial layer degradation and reduced AAA wall tissue content. The mismatch in material compliance between calcification and wall tissue leads to local high stress concentration in adjacent tissue areas, ultimately increasing the risk of AAA rupture (97, 98). The specific mechanism still needs to be further explored.

Furthermore, angiotensin-converting enzyme inhibitors (ACEIs) and angiotensin receptor blockers (ARBs), two common antihypertensive agents, have been found to be inversely associated with AAA mortality (58). Telmisartan, an angiotensin-converting enzyme inhibitor known for its antihypertensive properties, has been demonstrated to prevent the development of experimental AAA by inhibiting the angiotensin II type 1a (AT1a) receptor. However, the clinical treatment effect of telmisartan for small AAA is not significant (99, 100).

### 4.2 Obesity paradox caused by obesity evaluation index—BMI paradox

BMI has traditionally served as a readily accessible but imperfect surrogate for obesity. With only height and weight considered, BMI fails to capture the impact of central obesity on cardiovascular disease, diminishing its utility as a measure of obesity (101, 102). Indeed, studies have indicated that obesity, as represented by BMI, exhibits no significant association with AAA-related morbidity and is even inversely associated with AAA mortality (12, 87).However, when BMI is further dissected into fat and fat-free mass index, a negative correlation between fat-free mass and AAA morbidity emerges, highlighting the inadequacy of BMI alone in describing adipose tissue mass, distribution, and function (12). As the understanding of obesity deepens and imaging technology advances, more accurate measures of obesity have emerged, such as physical activity, physical activity, VAT area, PVAT density, and epicardial adipose tissue volume (EATV) (16, 103). Physical activity is known to improve cardiovascular health, reduce inflammation, and enhance metabolic profiles, all of which can positively impact the progression and management of AAA (77). While physical activity contributes to maintaining a healthy weight and reducing visceral fat, studies have noted that accurately quantifying physical activity levels can be challenging, and self-reported activity may not always reflect true levels. Additionally, cardiorespiratory fitness (CRF) is frequently mentioned as an important metric for assessing obesity. CRF has been demonstrated to be a robust indicator of overall cardiovascular health and is often a better predictor of mortality and morbidity than BMI (104). High CRF levels are associated with improved vascular function, lower inflammation, and reduced cardiovascular risk. Visceral adipose tissue (VAT) and perivascular adipose tissue (PVAT) exert a greater and more direct impact on the cardiovascular system compared to subcutaneous adipose tissue, which has been shown to have minimal influence on cardiovascular disease (105). Future studies should aim to integrate these indices to evaluate their combined impact on the progression and management of AAA. This approach can help to identify at-risk individuals more accurately and develop tailored intervention strategies that address both weight management and overall cardiovascular fitness.

## 5 Weight loss and prehabilitation in AAA

It has been reported that severely obese patients undergoing AAA repair experience higher postoperative mortality, longer average hospital stays, and increased total hospital costs, primarily attributed to thromboembolism, wound infection, and renal complications (24, 106). Based on the modified Johns Hopkins surgical criteria, aortic surgery is considered high-risk with a cardiac risk exceeding 5% (107). During open aortic repair (OAR), excess fat can lead to challenges such as increased incision length, surgical dissection difficulty, prolonged surgery and anesthesia duration, and the need for postoperative ventilatory support, all of which elevate the risk of surgery and contribute to higher perioperative mortality (108). Although mortality rates do not increase with EVAR, severely obese patients face a higher risk of stroke and wound complications consistent with those observed in OAR (109). Furthermore, prolonged surgical incisions and excess fat can impede wound healing, potentially leading to graft infection, sepsis, and fatal outcomes (78).

Preoperative weight loss in severely obese patients has been shown to reduce perioperative risk. Similar to other cardiovascular diseases, moderate preoperative physical activity in AAA patients is beneficial, reducing the risk of cardiac, renal, and respiratory complications while maintaining safety (110, 111). Indeed, preoperative weight loss can be considered a form of prehabilitation, enhancing cardiopulmonary function reserve and positively impacting postoperative recovery. Studies have demonstrated that Angiotensin II-induced AAA mice fed a low-fat diet exhibit reduced abdominal aortic diameter and neovascularization compared to those fed a high-fat diet (112).

Moreover, a prospective randomized controlled trial involving 56 AAA patients revealed that participation in a community exercise program significantly improved peak oxygen consumption (VO2) starting at week 16 and also enhanced triglyceride levels and health-related quality of life, significantly impacting perioperative mortality of AAA (113). Additionally, an interventional study involving 144 overweight and obese patients showed that weight loss induced by calorie restriction may reduce both thoracic and abdominal aortic diameters, although further investigation is required to ascertain their impact on AAA development prevention (114). However, a meta-analysis has indicated that prehabilitation exercise therapy may not reduce perioperative complications or length of hospital stay in AAA patients (115). This discrepancy could be attributed to the lack of separate comparisons among patients with different weight levels.

In addition to the aforementioned preoperative physical activity, medical interventions targeting severe obesity have been shown to reduce perioperative mortality in AAA. Several medical interventions aimed at treating obesity have shown promise in mitigating the progression of AAA. Pharmacological treatments, such as the use of statins, have been associated with both weight reduction and a decrease in the progression of AAA. Statins, known for their lipid-lowering effects, also possess anti-inflammatory properties that can help reduce the chronic inflammation associated with both obesity and AAA (116).Additionally, medications such as antihypertensives and antidiabetic agents may indirectly influence AAA progression by controlling blood pressure and glucose levels, thereby reducing vascular stress and inflammation (117). Despite substantial evidence indicating that bariatric surgeries, including gastric bypass and sleeve gastrectomy, can significantly reduce obesity-related perioperative mortality, there is a lack of specific reports on AAA, possibly due to issues related to the timing of surgery (118).

Building upon this understanding, we hypothesize that severe obese patients with AAA, who do not currently meet surgical criteria, can achieve a state of mild obesity through guided physical activity and diet control overseen by healthcare professionals. This approach could potentially reduce perioperative complications and delay the progression to surgical intervention, thus alleviating the economic burden on society.

Admittedly, this hypothesis warrants further investigation through both retrospective and prospective studies to assess its safety and feasibility. Such studies would provide valuable guidance in determining the efficacy and potential benefits of implementing physical activity and medical interventions in severe obese patients with AAA.

## 6 Discussion

This review comprehensively explores the relationship between obesity and AAA morbidity, perioperative mortality, and delves into the obesity paradox within AAA disease, analyzing its multifaceted causes. It emphasizes that both AAA and obesity are chronic aging diseases, shedding light on their intricate interplay. Furthermore, the review briefly discusses and looks ahead to the potential of preoperative prehabilitation for AAA patients.

AAA has long been conceptualized as a manifestation of aortic aging, characterized by various histological changes. With advancing age, the aorta undergoes processes such as endothelial cell apoptosis, a switch in smooth muscle contraction phenotype, inflammation infiltration, and elastin degeneration (8, 119). At the cytological level, clinical studies have indicated decreased telomerase expression in the aortic endothelial nucleus of AAA patients compared to non-AAA individuals (120). Moreover, lysosome autophagy function is compromised in AAA. The TFEB gene, a crucial regulator of autophagy and lysosomal biogenesis, plays a pivotal role in maintaining cellular homeostasis. Experimental evidence has shown that TFEB gene knockdown in mouse VSMCs exacerbates the progression of experimental AAA (121).

In addition, several anti-aging drugs, including spermidine and polyunsaturated fatty acids, have demonstrated protective effects against the occurrence and progression of AAA in animal models by mitigating inflammatory infiltration, modulating autophagy, and preserving smooth muscle contraction phenotype (122, 123). However, randomized controlled clinical trials have yielded inconclusive results regarding the efficacy of polyunsaturated fatty acids in inhibiting or reversing the progression of AAAs. Despite their potential to improve the fatty acid profile and reduce vascular inflammatory infiltration and arterial stiffness in AAA patients, there remains insufficient evidence to support their role in halting or reversing AAA progression (124, 125).

Furthermore, adipocyte accumulation, particularly visceral fat associated with central obesity, contributes to systemic aging by promoting the secretion of inflammatory cytokines and altering the secretion of adipokines. This aging effect extends beyond blood vessels to affect other physiological systems, including the musculoskeletal and metabolic systems (126–128). Therefore, obesity can be viewed as a form of systemic aging.

Interestingly, the degree of obesity does not exhibit a linear correlation with perioperative mortality in AAA patients, despite representing systemic aging. This phenomenon, known as the obesity paradox, is not unique to AAA but is also observed in various other cardiovascular and non-cardiovascular diseases, such as coronary heart disease, cancer, and COPD (129–132). For instance, a randomized controlled clinical trial involving patients with hypertension and coronary heart disease revealed that individuals with a BMI of 25-30 kg/m2 had the lowest perioperative risk and risk of death compared to normal-weight patients (131). Similarly, a meta-analysis involving patients with colorectal cancer demonstrated that overweight patients had better overall, disease-free, and cancer-specific survival than normal-weight patients (129).

The obesity paradox is predominantly observed in chronic diseases and is more prevalent among the elderly population. This phenomenon may stem from the complex interplay between chronic disease consumption and aging-related changes in the body, leading to decreased protein content, immune system disorders, weakened resistance, and increased mortality (133, 134). Notably, observational studies have shown that malnutrition and cachexia result in poor long-term survival after EVAR despite reductions in total body fat (135–137). This suggests that the essence of the obesity paradox lies not solely in total body adipose content but in the overall aging condition of the body. Additionally, adipose tissue can serve as an energy source for chronic disease consumption in patients with poor general body condition, providing theoretical support for perioperative prehabilitation strategies.

While weight loss methods such as physical activity and medical interventions have demonstrated benefits in reducing abdominal aorta diameter and improving perioperative outcomes, there is currently insufficient high-level evidence to support its effectiveness in reducing perioperative complications of AAA. This lack of evidence is reflected in guidelines such as those from the National Institute for Health and Care Excellence (NICE) (9, 113, 115). Therefore, the appropriate population for prehabilitation interventions targeting weight loss in AAA patients requires further clarification.

Given the significant role of obesity in the development, progression, and treatment of abdominal aortic aneurysms (AAA), it is crucial to develop screening, monitoring, and intervention strategies tailored to the varying degrees of obesity in patients at risk for AAA. Firstly, current guidelines could be enhanced by incorporating obesity-specific risk factors, such as body mass index (BMI) and waist circumference, to identify high-risk individuals earlier. Advanced imaging techniques, such as ultrasound and CT angiography, should be considered for regular monitoring of obese patients with known AAA. Secondly, regular assessments of inflammatory markers (e.g., CRP, IL-6) and metabolic parameters (e.g., blood glucose, lipid profiles) can provide insights into the progression of AAA and the effectiveness of intervention strategies. Wearable technology and telemedicine can also play a role in improving the frequency and convenience of monitoring for patients. Lastly, medical interventions, such as the use of statins and antihypertensive drugs, can help control underlying risk factors. For patients with severe obesity, bariatric surgery should be considered not only for weight loss but also for its potential benefits in reducing perioperative risks and inflammation associated with AAA. Furthermore, lifestyle interventions, including diet and exercise programs, should be customized to ensure long-term adherence and effectiveness.

**Figure 2:**
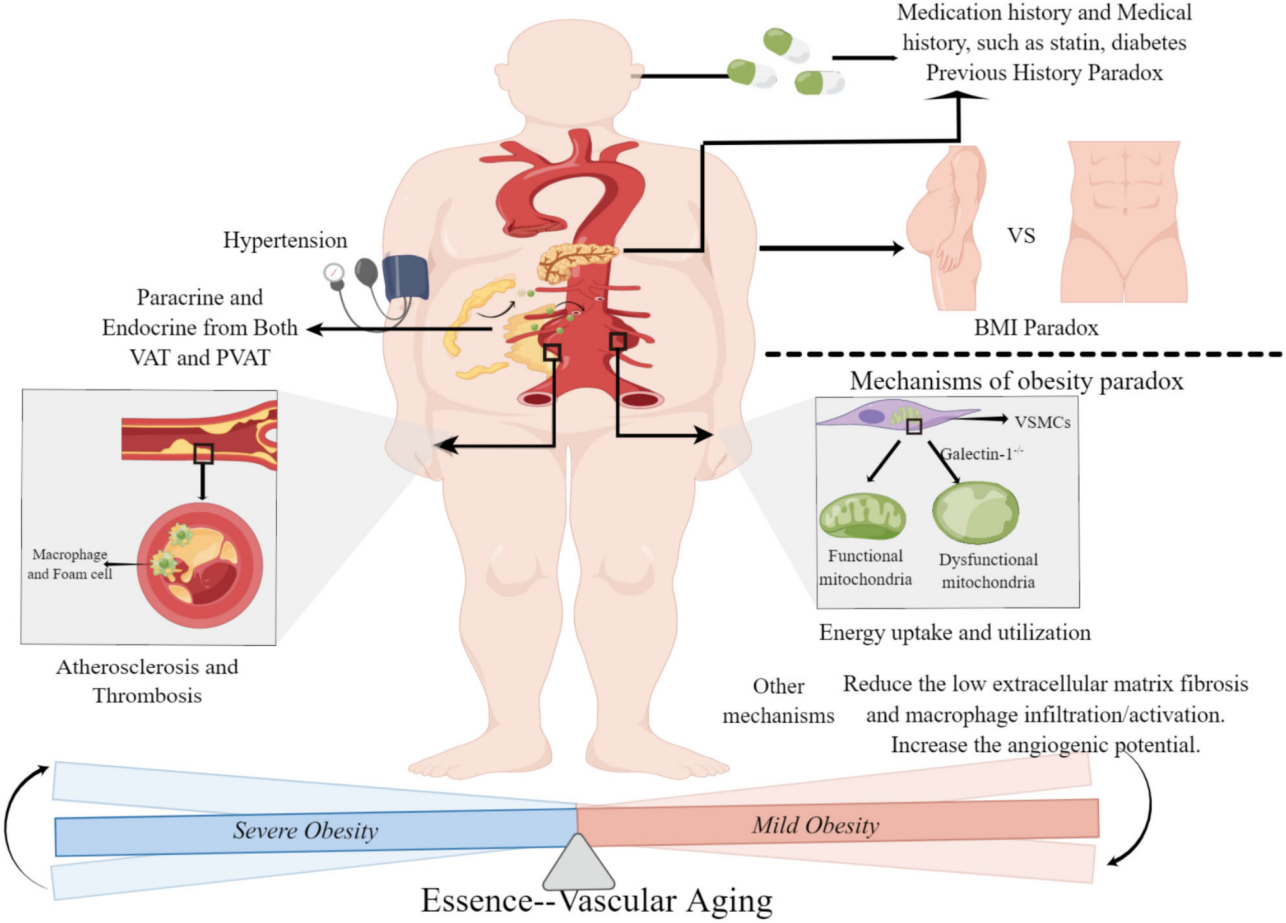
Obesity paradox in abdominal aortic aneurysm. Severe obesity leads to a high morbidity and mortality of abdominal aortic aneurysm through the endocrine/paracrine function of adipose tissue and obesity-related comorbidities and complications. Mild obese patients with abdominal aortic aneurysm have a better prognosis, mainly because of improved energy uptake and utilization and regulation of the perivascular environment. The main difference between severe obesity and mild obesity is the degree of systemic aging, and the effect of obesity on abdominal aortic aneurysm is essentially the embodiment of systemic aging in vascular disease. BMI, Body Mass Index; VAT, Visceral Adipose Tissue; PVAT, Perivascular Adipose Tissue. (This figure is supported by Figdraw).

Additionally, the methods and strategies for achieving weight loss in patients with AAA need to be further explored. It is essential to strike a balance between reducing the risk of AAA rupture and facilitating rapid rehabilitation. This balance poses an urgent problem that needs to be addressed through continued research and clinical practice. Future studies should focus on identifying effective and safe approaches to weight loss in AAA patients and evaluating their impact on perioperative complications and overall outcomes. Moreover, individualized approaches considering the unique characteristics and needs of AAA patients may be necessary to optimize outcomes while minimizing risks (**Figure 2**).

## 7 Conclusions

In summary, severe obesity is associated with an increased risk of developing AAA and contributes to higher perioperative mortality, largely due to its complications and endocrine effects. Conversely, mild obesity appears to be protective against AAA mortality. While these observations may seem contradictory, they likely stem from factors such as measurement limitations and patients’ medical histories. However, the notion of an obesity paradox in AAA is better understood as a reflection of systemic aging in abdominal aortic disease.

Furthermore, we discussed the significance of preoperative weight loss for severe obese patients with AAA, emphasizing the importance of timing and methodology in achieving optimal outcomes. Addressing obesity prior to surgery may help mitigate perioperative risks and improve overall prognosis. However, further research is needed to better understand the mechanisms underlying these relationships and to refine strategies for preoperative weight management in AAA patients.

## Author contributions

FL: Conceptualization, Data curation, Visualization, Writing – original draft, Writing – review & editing. YL: Conceptualization, Writing – original draft. JZ: Conceptualization, Data curation, Writing – review & editing. ZC: Conceptualization, Data curation, Writing – review & editing. YYL: Conceptualization, Data curation, Visualization, Writing – review & editing. MZ: Conceptualization, Data curation, Visualization, Writing – review & editing. LW: Conceptualization, Data curation, Funding acquisition, Supervision, Visualization, Writing – review & editing, Writing – original draft.

## References

[1] Jebeile H Kelly AS O’Malley G Baur LA. Obesity in children and adolescents: epidemiology, causes, assessment, and management. Lancet Diabetes Endocrinol. (2022) 10:351–65. doi: 10.1016/S2213-8587(22)00047-X PMC9831747 35248172

[2] Burki T. European Commission classifies obesity as a chronic disease. Lancet Diabetes Endocrinol. (2021) 9:418. doi: 10.1016/S2213-8587(21)00145-5 34087168

[3] Gebreab SZ Vandeleur CL Rudaz D Strippoli MF Gholam-Rezaee M Castelao E. Psychosocial stress over the lifespan, psychological factors, and cardiometabolic risk in the community. Psychosom Med. (2018) 80:628–39. doi: 10.1097/PSY.0000000000000621 29965943

[4] Pan XF Wang L Pan A. Epidemiology and determinants of obesity in China. Lancet Diabetes Endocrinol. (2021) 9:373–92. doi: 10.1016/S2213-8587(21)00045-0 34022156

[5] Adams KF Schatzkin A Harris TB Kipnis V Mouw T Ballard-Barbash R. Overweight, obesity, and mortality in a large prospective cohort of persons 50 to 71 years old. N Engl J Med. (2006) 355:763–78. doi: 10.1056/NEJMoa055643 16926275

[6] Dwyer-Lindgren L Freedman G Engell RE Fleming TD Lim SS Murray CJ. Prevalence of physical activity and obesity in US counties, 2001-2011: a road map for action. Popul Health Metr. (2013) 11:7. doi: 10.1186/1478-7954-11-7 23842197 PMC3718620

[7] De Schutter A Lavie CJ Milani RV. The impact of obesity on risk factors and prevalence and prognosis of coronary heart disease-the obesity paradox. Prog Cardiovasc Dis. (2014) 56:401–8. doi: 10.1016/j.pcad.2013.08.003 24438731

[8] Golledge J. Abdominal aortic aneurysm: update on pathogenesis and medical treatments. Nat Rev Cardiol. (2019) 16:225–42. doi: 10.1038/s41569-018-0114-9 30443031

[9] NICE guideline . Available online at: https://www.nice.org.uk/guidance/ng156.

[10] Allison MA Kwan K DiTomasso D Wright CM Criqui MH. The epidemiology of abdominal aortic diameter. J Vasc Surg. (2008) 48:121–7. doi: 10.1016/j.jvs.2008.02.031 18515037

[11] Waterhouse DF Cahill RA. Simple adaptation of current abdominal aortic aneurysm screening programs may address all-cause cardiovascular mortality: prospective observational cohort study. Am Heart J. (2008) 155:938–45. doi: 10.1016/j.ahj.2007.12.013 18440345

[12] Larsson SC Bäck M Rees JMB Mason AM Burgess S. Body mass index and body composition in relation to 14 cardiovascular conditions in UK Biobank: a Mendelian randomization study. Eur Heart J. (2020) 41:221–6. doi: 10.1093/eurheartj/ehz388 PMC6945523 31195408

[13] Golledge J Clancy P Jamrozik K Norman PE. Obesity, adipokines, and abdominal aortic aneurysm: Health in Men study. Circulation. (2007) 116:2275–9. doi: 10.1161/CIRCULATIONAHA.107.717926 17967974

[14] Thanassoulis G Massaro JM Corsini E Rogers I Schlett CL Meigs JB. Periaortic adipose tissue and aortic dimensions in the Framingham Heart Study. J Am Heart Assoc. (2012) 1:e000885. doi: 10.1161/JAHA.112.000885 23316310 PMC3540669

[15] Dias-Neto M Meekel JP van Schaik TG Hoozemans J Sousa-Nunes F Henriques-Coelho T. High density of periaortic adipose tissue in abdominal aortic aneurysm. Eur J Vasc Endovasc Surg. (2018) 56:663–71. doi: 10.1016/j.ejvs.2018.07.008 30115505

[16] Kawai Y Banno H Sato T Ikeda S Tsuruoka T Sugimoto M. Epicardial adipose tissue volume is associated with abdominal aortic aneurysm expansion. J Vasc Surg. (2022) 76:1253–60. doi: 10.1016/j.jvs.2022.04.032 35661742

[17] Hu G Ding N Wang Z Jin Z. The association of body composition with abdominal aortic aneurysm growth after endovascular aneurysm repair. Insights Imaging. (2022) 13:76. doi: 10.1186/s13244-022-01187-7 35467156 PMC9038972

[18] Locham S Rizwan M Dakour-Aridi H Faateh M Nejim B Malas M. Outcomes after elective abdominal aortic aneurysm repair in obese versus nonobese patients. J Vasc Surg. (2018) 68:1696–705. doi: 10.1016/j.jvs.2018.03.414 29887226

[19] Sen I Tenorio ER Pitcher G Mix D Marcondes GB Lima GBB. Effect of obesity on radiation exposure, quality of life scores, and outcomes of fenestrated-branched endovascular aortic repair of pararenal and thoracoabdominal aortic aneurysms. J Vasc Surg. (2021) 73:1156–66.e2. doi: 10.1016/j.jvs.2020.07.088 32853700

[20] Jonker FH Schlösser FJ Dewan M Huddle M Sergi M Dardik A. Influence of obesity on in-hospital and midterm outcomes after endovascular repair of abdominal aortic aneurysm. J Endovasc Ther. (2009) 16:302–9. doi: 10.1583/08-2646.1 19642786

[21] Johnson ON 3rd Sidawy AN Scanlon JM Walcott R Arora S Macsata RA. Impact of obesity on outcomes after open surgical and endovascular abdominal aortic aneurysm repair. J Am Coll Surg. (2010) 210:166–77. doi: 10.1016/j.jamcollsurg.2009.10.011 20113936

[22] Takada M Yamagishi K Tamakoshi A Iso H. Body mass index and mortality from aortic aneurysm and dissection. J Atheroscler Thromb. (2021) 28:338–48. doi: 10.5551/jat.57232 PMC8147012 32727971

[23] Liang TW Wang SK Dimusto PD McAninch CM Acher CW Timsina LR. Association between body mass index and perioperative mortality after repair of ruptured abdominal aortic aneurysms. Vasc Endovascular Surg. (2020) 54:573–8. doi: 10.1177/1538574420939356 32643559

[24] Giles KA Wyers MC Pomposelli FB Hamdan AD Ching YA Schermerhorn ML. The impact of body mass index on perioperative outcomes of open and endovascular abdominal aortic aneurysm repair from the National Surgical Quality Improvement Program, 2005-2007. J Vasc Surg. (2010) 52:1471–7. doi: 10.1016/j.jvs.2010.07.013 PMC3005989 20843627

[25] Sidloff D Stather P Dattani N Bown M Thompson J Sayers R. Aneurysm global epidemiology study: public health measures can further reduce abdominal aortic aneurysm mortality. Circulation. (2014) 129:747–53. doi: 10.1161/CIRCULATIONAHA.113.005457 24249717

[26] Davenport DL Xenos ES Hosokawa P Radford J Henderson WG Endean ED. The influence of body mass index obesity status on vascular surgery 30-day morbidity and mortality. J Vasc Surg. (2009) 49:140–7, 7.e1. doi: 10.1016/j.jvs.2008.08.052 19028047

[27] Bellamkonda KS Scali ST D’Oria M Columbo JA Stableford J Goodney PP. The contemporary impact of body mass index on open aortic aneurysm repair. J Vasc Surg. (2023) S0741-5214(23)00081-2. doi: 10.1016/j.jvs.2023.01.019 36682598

[28] De Schutter A Kachur S Lavie CJ Boddepalli RS Patel DA Milani RV. The impact of inflammation on the obesity paradox in coronary heart disease. Int J Obes (Lond). (2016) 40:1730–5. doi: 10.1038/ijo.2016.125 27453423

[29] Horwich TB Fonarow GC Clark AL. Obesity and the obesity paradox in heart failure. Prog Cardiovasc Dis. (2018) 61:151–6. doi: 10.1016/j.pcad.2018.05.005 29852198

[30] Galyfos G Geropapas GI Kerasidis S Sianou A Sigala F Filis K. The effect of body mass index on major outcomes after vascular surgery. J Vasc Surg. (2017) 65:1193–207. doi: 10.1016/j.jvs.2016.09.032 27876519

[31] Iliodromiti S Celis-Morales CA Lyall DM Anderson J Gray SR Mackay DF. The impact of confounding on the associations of different adiposity measures with the incidence of cardiovascular disease: a cohort study of 296 535 adults of white European descent. Eur Heart J. (2018) 39:1514–20. doi: 10.1093/eurheartj/ehy057 PMC5930252 29718151

[32] Candow DG Chilibeck PD. Differences in size, strength, and power of upper and lower body muscle groups in young and older men. J Gerontol A Biol Sci Med Sci. (2005) 60:148–56. doi: 10.1093/gerona/60.2.148 15814855

[33] Tchernof A Després JP. Pathophysiology of human visceral obesity: an update. Physiol Rev. (2013) 93:359–404. doi: 10.1152/physrev.00033.2011 23303913

[34] Ungvari Z Tarantini S Donato AJ Galvan V Csiszar A. Mechanisms of vascular aging. Circ Res. (2018) 123:849–67. doi: 10.1161/CIRCRESAHA.118.311378 PMC6248882 30355080

[35] Summers KL Kerut EK Sheahan CM Sheahan MG 3rd. Evaluating the prevalence of abdominal aortic aneurysms in the United States through a national screening database. J Vasc Surg. (2021) 73:61–8. doi: 10.1016/j.jvs.2020.03.046 32330595

[36] Li SR Reitz KM Kennedy J Gabriel L Phillips AR Shireman PK. Epidemiology of age-, sex-, and race-specific hospitalizations for abdominal aortic aneurysms highlights gaps in current screening recommendations. J Vasc Surg. (2022) 76(5):1216–26.e4. doi: 10.1016/j.jvs.2022.02.058 PMC9458770 35278654

[37] Parvizi M Franchi F Arendt BK Ebtehaj S Rodriguez-Porcel M Lanza IR. Senolytic agents lessen the severity of abdominal aortic aneurysm in aged mice. Exp Gerontol. (2021) 151:111416. doi: 10.1016/j.exger.2021.111416 34022272 PMC11443445

[38] Teti G Chiarini F Mazzotti E Ruggeri A Carano F Falconi M. Cellular senescence in vascular wall mesenchymal stromal cells, a possible contribution to the development of aortic aneurysm. Mech Ageing Dev. (2021) 197:111515. doi: 10.1016/j.mad.2021.111515 34062172

[39] Parvizi M Ryan ZC Ebtehaj S Arendt BK Lanza IR. The secretome of senescent preadipocytes influences the phenotype and function of cells of the vascular wall. Biochim Biophys Acta Mol Basis Dis. (2021) 1867:165983. doi: 10.1016/j.bbadis.2020.165983 33002577

[40] Saxton SN Clark BJ Withers SB Eringa EC Heagerty AM. Mechanistic links between obesity, diabetes, and blood pressure: role of perivascular adipose tissue. Physiol Rev. (2019) 99:1701–63. doi: 10.1152/physrev.00034.2018 31339053

[41] Pouliot MC Després JP Lemieux S Moorjani S Bouchard C Tremblay A. Waist circumference and abdominal sagittal diameter: best simple anthropometric indexes of abdominal visceral adipose tissue accumulation and related cardiovascular risk in men and women. Am J Cardiol. (1994) 73:460–8. doi: 10.1016/0002-9149(94)90676-9 8141087

[42] Stackelberg O Björck M Sadr-Azodi O Larsson SC Orsini N Wolk A. Obesity and abdominal aortic aneurysm. Br J Surg. (2013) 100:360–6. doi: 10.1002/bjs.8983 23203847

[43] Schlett CL Massaro JM Lehman SJ Bamberg F O’Donnell CJ Fox CS. Novel measurements of periaortic adipose tissue in comparison to anthropometric measures of obesity, and abdominal adipose tissue. Int J Obes (Lond). (2009) 33:226–32. doi: 10.1038/ijo.2008.267 PMC3779879 19139753

[44] Mladenov M Lubomirov L Grisk O Avtanski D Mitrokhin V Sazdova I. Oxidative stress, reductive stress and antioxidants in vascular pathogenesis and aging. Antioxidants (Basel). (2023) 12(5):1126. doi: 10.3390/antiox12051126 37237992 PMC10215600

[45] Chen HZ Wang F Gao P Pei JF Liu Y Xu TT. Age-associated sirtuin 1 reduction in vascular smooth muscle links vascular senescence and inflammation to abdominal aortic aneurysm. Circ Res. (2016) 119:1076–88. doi: 10.1161/CIRCRESAHA.116.308895 PMC6546422 27650558

[46] Jabłońska A Zagrapan B Neumayer C Eilenberg W Scheuba A Brostjan C. Polymorphisms in the IL-6 and TNF-α gene are associated with an increased risk of abdominal aortic aneurysm. Int J Cardiol. (2021) 329:192–7. doi: 10.1016/j.ijcard.2020.12.051 33359288

[47] Thanigaimani S Golledge J. Role of adipokines and perivascular adipose tissue in abdominal aortic aneurysm: A systematic review and meta-analysis of animal and human observational studies. Front Endocrinol (Lausanne). (2021) 12:618434. doi: 10.3389/fendo.2021.618434 33796069 PMC8008472

[48] Tilg H Moschen AR. Adipocytokines: mediators linking adipose tissue, inflammation and immunity. Nat Rev Immunol. (2006) 6:772–83. doi: 10.1038/nri1937 16998510

[49] Horimatsu T Kim HW Weintraub NL. The role of perivascular adipose tissue in non-atherosclerotic vascular disease. Front Physiol. (2017) 8:969. doi: 10.3389/fphys.2017.00969 29234289 PMC5712360

[50] Zhang Y Chua S Jr. Leptin function and regulation. Compr Physiol. (2017) 8:351–69. doi: 10.1002/cphy.c160041 29357132

[51] Liu CL Ren J Wang Y Zhang X Sukhova GK Liao M. Adipocytes promote interleukin-18 binding to its receptors during abdominal aortic aneurysm formation in mice. Eur Heart J. (2020) 41:2456–68. doi: 10.1093/eurheartj/ehz856 PMC8453281 31821481

[52] Schroeter MR Eschholz N Herzberg S Jerchel I Leifheit-Nestler M Czepluch FS. Leptin-dependent and leptin-independent paracrine effects of perivascular adipose tissue on neointima formation. Arterioscler Thromb Vasc Biol. (2013) 33:980–7. doi: 10.1161/ATVBAHA.113.301393 23520165

[53] Ketonen J Shi J Martonen E Mervaala E. Periadventitial adipose tissue promotes endothelial dysfunction via oxidative stress in diet-induced obese C57Bl/6 mice. Circ J. (2010) 74:1479–87. doi: 10.1253/circj.CJ-09-0661 20526041

[54] Nava E Llorens S. The local regulation of vascular function: from an inside-outside to an outside-inside model. Front Physiol. (2019) 10:729. doi: 10.3389/fphys.2019.00729 31244683 PMC6581701

[55] Neves KB Lobato NS Lopes RA Filgueira FP Zanotto CZ Oliveira AM. Chemerin reduces vascular nitric oxide/cGMP signalling in rat aorta: a link to vascular dysfunction in obesity? Clin Sci (Lond). (2014) 127:111–22. doi: 10.1042/CS20130286 24498891

[56] Neves KB Nguyen Dinh Cat A Lopes RA Rios FJ Anagnostopoulou A Lobato NS. Chemerin regulates crosstalk between adipocytes and vascular cells through nox. Hypertension. (2015) 66:657–66. doi: 10.1161/HYPERTENSIONAHA.115.05616 26150435

[57] Chen S Han C Bian S Chen J Feng X Li G. Chemerin-9 attenuates experimental abdominal aortic aneurysm formation in apoE(-/-) mice. J Oncol. (2021) 2021:6629204. doi: 10.1155/2021/6629204 33953746 PMC8068550

[58] Kristensen KE Torp-Pedersen C Gislason GH Egfjord M Rasmussen HB Hansen PR. Angiotensin-converting enzyme inhibitors and angiotensin II receptor blockers in patients with abdominal aortic aneurysms: nation-wide cohort study. Arterioscler Thromb Vasc Biol. (2015) 35:733–40. doi: 10.1161/ATVBAHA.114.304428 25633315

[59] Zhang ZB Ruan CC Lin JR Xu L Chen XH Du YN. Perivascular adipose tissue-derived PDGF-D contributes to aortic aneurysm formation during obesity. Diabetes. (2018) 67:1549–60. doi: 10.2337/db18-0098 29794241

[60] Yang XF Wang H Huang Y Huang JH Ren HL Xu Q. Myeloid angiotensin II type 1 receptor mediates macrophage polarization and promotes vascular injury in DOCA/salt hypertensive mice. Front Pharmacol. (2022) 13:879693. doi: 10.3389/fphar.2022.879693 35721173 PMC9204513

[61] Ben-Zvi D Savion N Kolodgie F Simon A Fisch S Schäfer K. Local application of leptin antagonist attenuates angiotensin II-induced ascending aortic aneurysm and cardiac remodeling. J Am Heart Assoc. (2016) 5(5):003474. doi: 10.1161/JAHA.116.003474 PMC4889208 27143353

[62] Tanaka H Zaima N Sasaki T Hayasaka T Goto-Inoue N Onoue K. Adventitial vasa vasorum arteriosclerosis in abdominal aortic aneurysm. PloS One. (2013) 8:e57398. doi: 10.1371/journal.pone.0057398 23460850 PMC3583902

[63] Tanaka H Zaima N Sasaki T Sano M Yamamoto N Saito T. Hypoperfusion of the adventitial vasa vasorum develops an abdominal aortic aneurysm. PloS One. (2015) 10:e0134386. doi: 10.1371/journal.pone.0134386 26308526 PMC4550325

[64] Yang Y Xian W Wu D Huo Z Hong S Li Y. The role of obesity, type 2 diabetes, and metabolic factors in gout: A Mendelian randomization study. Front Endocrinol (Lausanne). (2022) 13:917056. doi: 10.3389/fendo.2022.917056 35992130 PMC9388832

[65] Kahn HS. The “lipid accumulation product” performs better than the body mass index for recognizing cardiovascular risk: a population-based comparison. BMC Cardiovasc Disord. (2005) 5:26. doi: 10.1186/1471-2261-5-26 16150143 PMC1236917

[66] Kubota Y Folsom AR Ballantyne CM Tang W. Lipoprotein(a) and abdominal aortic aneurysm risk: The Atherosclerosis Risk in Communities study. Atherosclerosis. (2018) 268:63–7. doi: 10.1016/j.atherosclerosis.2017.10.017 PMC5788200 29182987

[67] Nastasi DR Norman R Moxon JV Quigley F Velu R Jenkins J. The potential benefits and costs of an intensified approach to low density lipoprotein cholesterol lowering in people with abdominal aortic aneurysm. Eur J Vasc Endovasc Surg. (2021) 62:643–50. doi: 10.1016/j.ejvs.2021.06.031 34507892

[68] Hoeke G Kooijman S Boon MR Rensen PC Berbée JF. Role of brown fat in lipoprotein metabolism and atherosclerosis. Circ Res. (2016) 118:173–82. doi: 10.1161/CIRCRESAHA.115.306647 26837747

[69] Virmani R Burke AP Farb A Kolodgie FD. Pathology of the vulnerable plaque. J Am Coll Cardiol. (2006) 47:C13–8. doi: 10.1016/j.jacc.2005.10.065 16631505

[70] Henry SL Barzel B Wood-Bradley RJ Burke SL Head GA Armitage JA. Developmental origins of obesity-related hypertension. Clin Exp Pharmacol Physiol. (2012) 39:799–806. doi: 10.1111/j.1440-1681.2011.05579.x 21801195

[71] Rasouli N Kern PA. Adipocytokines and the metabolic complications of obesity. J Clin Endocrinol Metab. (2008) 93:S64–73. doi: 10.1210/jc.2008-1613 PMC2585759 18987272

[72] Mark AL. Selective leptin resistance revisited. Am J Physiol Regul Integr Comp Physiol. (2013) 305:R566–81. doi: 10.1152/ajpregu.00180.2013 PMC3763044 23883674

[73] Sakalihasan N Michel JB Katsargyris A Kuivaniemi H Defraigne JO Nchimi A. Abdominal aortic aneurysms. Nat Rev Dis Primers. (2018) 4:34. doi: 10.1038/s41572-018-0030-7 30337540

[74] Boyd AJ Kuhn DC Lozowy RJ Kulbisky GP. Low wall shear stress predominates at sites of abdominal aortic aneurysm rupture. J Vasc Surg. (2016) 63:1613–9. doi: 10.1016/j.jvs.2015.01.040 25752691

[75] Brown IAM Diederich L Good ME DeLalio LJ Murphy SA Cortese-Krott MM. Vascular smooth muscle remodeling in conductive and resistance arteries in hypertension. Arterioscler Thromb Vasc Biol. (2018) 38:1969–85. doi: 10.1161/ATVBAHA.118.311229 PMC6205219 30354262

[76] Aune D Schlesinger S Norat T Riboli E. Tobacco smoking and the risk of abdominal aortic aneurysm: a systematic review and meta-analysis of prospective studies. Sci Rep. (2018) 8:14786. doi: 10.1038/s41598-018-32100-2 30283044 PMC6170425

[77] Aune D Sen A Kobeissi E Hamer M Norat T Riboli E. Physical activity and the risk of abdominal aortic aneurysm: a systematic review and meta-analysis of prospective studies. Sci Rep. (2020) 10:22287. doi: 10.1038/s41598-020-76306-9 33339835 PMC7749100

[78] Naiem AA Kim AY Mahmoud I Gill HL. A systematic review and meta-analysis evaluating the impact of obesity on outcomes of abdominal aortic aneurysm treatment. J Vasc Surg. (2022) 75:1450–5.e3. doi: 10.1016/j.jvs.2021.10.053 34785300

[79] Gaber T Strehl C Buttgereit F. Metabolic regulation of inflammation. Nat Rev Rheumatol. (2017) 13:267–79. doi: 10.1038/nrrheum.2017.37 28331208

[80] Lavie CJ De Schutter A Parto P Jahangir E Kokkinos P Ortega FB. Obesity and prevalence of cardiovascular diseases and prognosis-the obesity paradox updated. Prog Cardiovasc Dis. (2016) 58:537–47. doi: 10.1016/j.pcad.2016.01.008 26826295

[81] De Rosa M Gambardella J Shu J Santulli G. Dietary fat is a key determinant in balancing mitochondrial dynamics in heart failure: a novel mechanism underlying the obesity paradox. Cardiovasc Res. (2018) 114:925–7. doi: 10.1093/cvr/cvy074 PMC5967494 29579150

[82] Baek JH Kim DH Lee J Kim SJ Chun KH. Galectin-1 accelerates high-fat diet-induced obesity by activation of peroxisome proliferator-activated receptor gamma (PPARγ) in mice. Cell Death Dis. (2021) 12:66. doi: 10.1038/s41419-020-03367-z 33431823 PMC7801586

[83] Roldán-Montero R Pérez-Sáez JM Cerro-Pardo I Oller J Martinez-Lopez D Nuñez E. Galectin-1 prevents pathological vascular remodeling in atherosclerosis and abdominal aortic aneurysm. Sci Adv. (2022) 8:eabm7322. doi: 10.1126/sciadv.abm7322 35294231 PMC8926342

[84] Antonopoulos AS Tousoulis D. The molecular mechanisms of obesity paradox. Cardiovasc Res. (2017) 113:1074–86. doi: 10.1093/cvr/cvx106 28549096

[85] Holmes MV Lange LA Palmer T Lanktree MB North KE Almoguera B. Causal effects of body mass index on cardiometabolic traits and events: a Mendelian randomization analysis. Am J Hum Genet. (2014) 94:198–208. doi: 10.1016/j.ajhg.2013.12.014 24462370 PMC3928659

[86] Fall T Hägg S Mägi R Ploner A Fischer K Horikoshi M. The role of adiposity in cardiometabolic traits: a Mendelian randomization analysis. PloS Med. (2013) 10:e1001474. doi: 10.1371/journal.pmed.1001474 23824655 PMC3692470

[87] Png CYM Wu J Tang TY Png IPL Sheng TJ Choke E. Editor’s choice - decrease in mortality from abdominal aortic aneurysms (2001 to 2015): is it decreasing even faster? Eur J Vasc Endovasc Surg. (2021) 61:900–7. doi: 10.1016/j.ejvs.2021.02.013 33773903

[88] Raffort J Lareyre F Clément M Hassen-Khodja R Chinetti G Mallat Z. Diabetes and aortic aneurysm: current state of the art. Cardiovasc Res. (2018) 114:1702–13. doi: 10.1093/cvr/cvy174 PMC6198737 30052821

[89] Xiong J Wu Z Chen C Wei Y Guo W. Association between diabetes and prevalence and growth rate of abdominal aortic aneurysms: A meta-analysis. Int J Cardiol. (2016) 221:484–95. doi: 10.1016/j.ijcard.2016.07.016 27414727

[90] Dinesh Shah A Langenberg C Rapsomaniki E Denaxas S Pujades-Rodriguez M Gale CP. Type 2 diabetes and incidence of a wide range of cardiovascular diseases: a cohort study in 1·9 million people. Lancet. (2015) 385 Suppl 1:S86. doi: 10.1016/j.ejvs.2021.02.013 26312908

[91] Hinchliffe RJ. Metformin and abdominal aortic aneurysm. Eur J Vasc Endovasc Surg. (2017) 54:679–80. doi: 10.1016/j.ejvs.2017.08.016 28988609

[92] Golledge J Moxon J Pinchbeck J Anderson G Rowbotham S Jenkins J. Association between metformin prescription and growth rates of abdominal aortic aneurysms. Br J Surg. (2017) 104:1486–93. doi: 10.1002/bjs.10587 28650557

[93] Zhao P Sui BD Liu N Lv YJ Zheng CX Lu YB. Anti-aging pharmacology in cutaneous wound healing: effects of metformin, resveratrol, and rapamycin by local application. Aging Cell. (2017) 16:1083–93. doi: 10.1111/acel.12635 PMC5595695 28677234

[94] Klopf J Fuchs L Schernthaner R Domenig CM Gollackner B Brostjan C. The prognostic impact of vascular calcification on abdominal aortic aneurysm progression. J Vasc Surg. (2022) 75:1926–34. doi: 10.1016/j.jvs.2021.11.062 34921970

[95] Nakayama A Morita H Hayashi N Nomura Y Hoshina K Shigematsu K. Inverse correlation between calcium accumulation and the expansion rate of abdominal aortic aneurysms. Circ J. (2016) 80:332–9. doi: 10.1253/circj.CJ-15-1065 26639068

[96] Matthews EO Rowbotham SE Moxon JV Jones RE Vega de Ceniga M Golledge J. Meta-analysis of the association between peripheral artery disease and growth of abdominal aortic aneurysms. Br J Surg. (2017) 104:1765–74. doi: 10.1002/bjs.10675 29044481

[97] Barrett HE Cunnane EM Hidayat H O’Brien JM Moloney MA Kavanagh EG. On the influence of wall calcification and intraluminal thrombus on prediction of abdominal aortic aneurysm rupture. J Vasc Surg. (2018) 67:1234–46.e2. doi: 10.1016/j.jvs.2017.05.086 28899569

[98] Barrett HE Cunnane EM JM MA M EG K Walsh MT. On the effect of computed tomography resolution to distinguish between abdominal aortic aneurysm wall tissue and calcification: A proof of concept. Eur J Radiol. (2017) 95:370–7. doi: 10.1016/j.ejrad.2017.08.023 28987694

[99] Krueger F Kappert K Foryst-Ludwig A Kramer F Clemenz M Grzesiak A. AT1-receptor blockade attenuates outward aortic remodeling associated with diet-induced obesity in mice. Clin Sci (Lond). (2017) 131:1989–2005. doi: 10.1042/CS20170131 28646121

[100] Golledge J Pinchbeck J Tomee SM Rowbotham SE Singh TP Moxon JV. Efficacy of telmisartan to slow growth of small abdominal aortic aneurysms: A randomized clinical trial. JAMA Cardiol. (2020) 5:1374–81. doi: 10.1001/jamacardio.2020.3524 PMC7450408 32845283

[101] Romero-Corral A Somers VK Sierra-Johnson J Jensen MD Thomas RJ Squires RW. Diagnostic performance of body mass index to detect obesity in patients with coronary artery disease. Eur Heart J. (2007) 28:2087–93. doi: 10.1093/eurheartj/ehm243 17626030

[102] Poirier P. Adiposity and cardiovascular disease: are we using the right definition of obesity? Eur Heart J. (2007) 28:2047–8. doi: 10.1016/j.ejvs.2021.02.013 17673449

[103] Apoloni RC Zerati AE Wolosker N Saes GF Wolosker M Curado T. Analysis of the correlation between central obesity and abdominal aortic diseases. Ann Vasc Surg. (2019) 54:176–84. doi: 10.1016/j.avsg.2018.06.016 30103051

[104] Tew GA Batterham AM Colling K Gray J Kerr K Kothmann E. Randomized feasibility trial of high-intensity interval training before elective abdominal aortic aneurysm repair. Br J Surg. (2017) 104:1791–801. doi: 10.1002/bjs.10669 28990651

[105] Kissebah AH Krakower GR. Regional adiposity and morbidity. Physiol Rev. (1994) 74:761–811. doi: 10.1152/physrev.1994.74.4.761 7938225

[106] Khorgami Z Sclabas GM Aminian A Lau PJ Chow GS Malgor RD. Mortality in open abdominal aortic surgery in patients with morbid obesity. Surg Obes Relat Dis. (2019) 15:958–63. doi: 10.1016/j.soard.2019.03.044 31097382

[107] Donati A Ruzzi M Adrario E Pelaia P Coluzzi F Gabbanelli V. A new and feasible model for predicting operative risk. Br J Anaesth. (2004) 93:393–9. doi: 10.1093/bja/aeh210 15220171

[108] Dong D Peng X Liu J Qian H Li J Wu B. Morbid obesity alters both pharmacokinetics and pharmacodynamics of propofol: dosing recommendation for anesthesia induction. Drug Metab Dispos. (2016) 44:1579–83. doi: 10.1124/dmd.116.071605 27481855

[109] Saratzis A Saedon M Melas N Kitas GD Mahmood A. Obesity as an independent predictor of outcome after endovascular abdominal aortic aneurysm repair. Ann Vasc Surg. (2014) 28:816–22. doi: 10.1016/j.avsg.2013.07.008 24378248

[110] Wee IJY Choong A. A systematic review of the impact of preoperative exercise for patients with abdominal aortic aneurysm. J Vasc Surg. (2020) 71:2123–31.e1. doi: 10.1016/j.jvs.2018.09.039 30606665

[111] Kato M Kubo A Green FN Takagi H. Meta-analysis of randomized controlled trials on safety and efficacy of exercise training in patients with abdominal aortic aneurysm. J Vasc Surg. (2019) 69:933–43. doi: 10.1016/j.jvs.2018.07.069 30578072

[112] Police SB Putnam K Thatcher S Batifoulier-Yiannikouris F Daugherty A Cassis LA. Weight loss in obese C57BL/6 mice limits adventitial expansion of established angiotensin II-induced abdominal aortic aneurysms. Am J Physiol Heart Circ Physiol. (2010) 298:H1932–8. doi: 10.1152/ajpheart.00961.2009 PMC2886622 20304811

[113] Haque A Wisely N McCollum C. Editor’s choice - the abdominal aortic aneurysm get fit trial: A randomised controlled trial of exercise to improve fitness in patients with abdominal aortic aneurysm. Eur J Vasc Endovasc Surg. (2022) 64:309–19. doi: 10.1016/j.ejvs.2022.07.005 35853580

[114] Stoll S Sowah SA Fink MA Nonnenmacher T Graf ME Johnson T. Changes in aortic diameter induced by weight loss: The HELENA trial- whole-body MR imaging in a dietary intervention trial. Front Physiol. (2022) 13:976949. doi: 10.3389/fphys.2022.976949 36203934 PMC9531129

[115] Fenton C Tan AR Abaraogu UO McCaslin JE. Prehabilitation exercise therapy before elective abdominal aortic aneurysm repair. Cochrane Database Syst Rev. (2021) 7:Cd013662. doi: 10.12968/bjca.2023.0078 34236703 PMC8275457

[116] Xiong X Wu Z Qin X Huang Q Wang X Qin J. Meta-analysis suggests statins reduce mortality after abdominal aortic aneurysm repair. J Vasc Surg. (2022) 75:356–62.e4. doi: 10.1016/j.jvs.2021.06.033 34197945

[117] Bahia SS Vidal-Diez A Seshasai SR Shpitser I Brownrigg JR Patterson BO. Cardiovascular risk prevention and all-cause mortality in primary care patients with an abdominal aortic aneurysm. Br J Surg. (2016) 103:1626–33. doi: 10.1002/bjs.10269 27704527

[118] Arterburn DE Telem DA Kushner RF Courcoulas AP. Benefits and risks of bariatric surgery in adults: A review. Jama. (2020) 324:879–87. doi: 10.1001/jama.2020.12567 32870301

[119] Golledge J Muller J Daugherty A Norman P. Abdominal aortic aneurysm: pathogenesis and implications for management. Arterioscler Thromb Vasc Biol. (2006) 26:2605–13. doi: 10.1161/01.ATV.0000245819.32762.cb 16973970

[120] Dimitroulis D Katsargyris A Klonaris C Avgerinos ED Fragou-Plemenou M Kouraklis G. Telomerase expression on aortic wall endothelial cells is attenuated in abdominal aortic aneurysms compared to healthy nonaneurysmal aortas. J Vasc Surg. (2011) 54:1778–83. doi: 10.1016/j.jvs.2011.06.079 21917401

[121] Lu H Sun J Liang W Chang Z Rom O Zhao Y. Cyclodextrin prevents abdominal aortic aneurysm via activation of vascular smooth muscle cell transcription factor EB. Circulation. (2020) 142:483–98. doi: 10.1161/CIRCULATIONAHA.119.044803 PMC7606768 32354235

[122] Liu S Huang T Liu R Cai H Pan B Liao M. Spermidine suppresses development of experimental abdominal aortic aneurysms. J Am Heart Assoc. (2020) 9:e014757. doi: 10.1161/JAHA.119.014757 32308093 PMC7428527

[123] Yoshihara T Shimada K Fukao K Sai E Sato-Okabayashi Y Matsumori R. Omega 3 polyunsaturated fatty acids suppress the development of aortic aneurysms through the inhibition of macrophage-mediated inflammation. Circ J. (2015) 79:1470–8. doi: 10.1253/circj.CJ-14-0471 25925976

[124] Meital LT Windsor MT Ramirez Jewell RML Young P Schulze K Magee R. n-3 PUFAs improve erythrocyte fatty acid profile in patients with small AAA: a randomized controlled trial. J Lipid Res. (2019) 60:1154–63. doi: 10.1194/jlr.P093013 PMC6547637 30914500

[125] Meital LT Schulze K Magee R O’Donnell J Jha P Meital CY. Long chain omega-3 polyunsaturated fatty acids improve vascular stiffness in abdominal aortic aneurysm: A randomized controlled trial. Nutrients. (2020) 13(1):138. doi: 10.3390/nu13010138 33396567 PMC7824679

[126] Reyes-Farias M Fos-Domenech J Serra D Herrero L Sánchez-Infantes D. White adipose tissue dysfunction in obesity and aging. Biochem Pharmacol. (2021) 192:114723. doi: 10.1016/j.bcp.2021.114723 34364887

[127] Bartlett DE Miller RB Thiesfeldt S Lakhani HV Shapiro JI Sodhi K. The role of na/K-ATPase signaling in oxidative stress related to aging: implications in obesity and cardiovascular disease. Int J Mol Sci. (2018) 19(7):2139. doi: 10.3390/ijms19072139 30041449 PMC6073138

[128] Liu Z Wu KKL Jiang X Xu A Cheng KKY. The role of adipose tissue senescence in obesity- and ageing-related metabolic disorders. Clin Sci (Lond). (2020) 134:315–30. doi: 10.1042/CS20190966 31998947

[129] Li Y Li C Wu G Yang W Wang X Duan L. The obesity paradox in patients with colorectal cancer: a systematic review and meta-analysis. Nutr Rev. (2022) 80:1755–68. doi: 10.1093/nutrit/nuac005 35182150

[130] Pagidipati NJ Zheng Y Green JB McGuire DK Mentz RJ Shah S. Association of obesity with cardiovascular outcomes in patients with type 2 diabetes and cardiovascular disease: Insights from TECOS. Am Heart J. (2020) 219:47–57. doi: 10.1016/j.ahj.2019.09.016 31707324

[131] Uretsky S Messerli FH Bangalore S Champion A Cooper-Dehoff RM Zhou Q. Obesity paradox in patients with hypertension and coronary artery disease. Am J Med. (2007) 120:863–70. doi: 10.1016/j.amjmed.2007.05.011 17904457

[132] DeLapp DA Glick C Furmanek S Ramirez JA Cavallazzi R. Patients with obesity have better long-term outcomes after hospitalization for COPD exacerbation. Copd. (2020) 17:373–7. doi: 10.1080/15412555.2020.1781805 32586139

[133] Short KR Vittone JL Bigelow ML Proctor DN Nair KS. Age and aerobic exercise training effects on whole body and muscle protein metabolism. Am J Physiol Endocrinol Metab. (2004) 286:E92–101. doi: 10.1152/ajpendo.00366.2003 14506079

[134] Yousefzadeh MJ Flores RR Zhu Y Schmiechen ZC Brooks RW Trussoni CE. An aged immune system drives senescence and ageing of solid organs. Nature. (2021) 594:100–5. doi: 10.1038/s41586-021-03547-7 PMC8684299 33981041

[135] Ikeda S Kodama A Kawai Y Tsuruoka T Sugimoto M Niimi K. Preoperative sarcopenia and malnutrition are correlated with poor long-term survival after endovascular abdominal aortic aneurysm repair. Surg Today. (2022) 52:98–105. doi: 10.1007/s00595-021-02362-x 34477979

[136] LeBoff MS Chou SH Ratliff KA Cook NR Khurana B Kim E. Supplemental vitamin D and incident fractures in midlife and older adults. N Engl J Med. (2022) 387:299–309. doi: 10.1056/NEJMoa2202106 35939577 PMC9716639

[137] Collins PF Elia M Stratton RJ. Nutritional support and functional capacity in chronic obstructive pulmonary disease: a systematic review and meta-analysis. Respirology. (2013) 18:616–29. doi: 10.1111/resp.12070 23432923

